# Wallenda regulates JNK-mediated cell death in *Drosophila*

**DOI:** 10.1038/cddis.2015.111

**Published:** 2015-05-07

**Authors:** X Ma, W Xu, D Zhang, Y Yang, W Li, L Xue

**Affiliations:** 1 Institute of Intervention Vessel, Shanghai 10th People's Hospital, Shanghai Key Laboratory of Signaling and Disease Research, School of Life Science and Technology, Tongji University, Shanghai 200092, China

## Abstract

The c-Jun N-terminal kinase (JNK) pathway plays essential roles in regulating a variety of cellular processes including proliferation, migration and survival. Previous genetic studies in *Drosophila* have identified numerous cell death regulating genes, providing new insights into the mechanisms for related diseases. Despite the known role of the small GTPase Rac1 in regulating cell death, the downstream components and underlying mechanism remain largely elusive. Here, we show that Rac1 promotes JNK-dependent cell death through Wallenda (Wnd). In addition, we find that Wnd triggers JNK activation and cell death via its kinase domain. Moreover, we show that both MKK4 and Hep are critical for Wnd-induced cell death. Furthermore, Wnd is essential for ectopic Egr- or Rho1-induced JNK activation and cell death. Finally, Wnd is physiologically required for loss of *scribble*-induced JNK-dependent cell death. Thus, our data suggest that *wnd* encodes a novel essential cell death regulator in *Drosophila*.

Programmed cell death (PCD) is a fundamental biological process required for normal organ development and tissue homeostasis in multicellular organisms.^1^ Disruption of PCD would result in a variety of diseases including neurodegenerative diseases, autoimmune disorders and cancers.^2^ *Drosophila melanogaster*, with its well-established genetic techniques and compact genome size, has been regarded as an excellent model organism to study PCD and its related signaling pathways.^3, 4^ The c-Jun N-terminal kinase (JNK) signaling has been implicated as one of the most important pathways that regulates various fundamental cell behaviors, such as proliferation, migration and cell death.^5, 6^

Rac1 belongs to the Rho family of small GTPase that regulates many aspects of physiological activities ranging from immune response to wound healing and migration.^7, 8, 9, 10, 11^ For instance, Rac1 has been implicated in JNK-mediated dorsal closure via Slpr (Slipper) in fly,^7^ osteoclast differentiation through TAK1-mediated NF-*κ*B signaling^12^ and myocyte hypertrophy via Ask1 (apoptotic signal-regulating kinase 1) in mammals.^13^ However, despite the reported role of Rac1 in cell death,^14^ its underlying mechanism and downstream components remain largely elusive.

Here by using *Drosophila* compound eye as a model, we found Rac1 expression induces JNK-dependent cell death and identified Wallenda (Wnd), a MAPKKK (mitogen-activated protein kinase kinase kinase) member as an essential downstream mediator. Furthermore, we found that Wnd is sufficient to induce JNK-mediated cell death through both Hep and MKK4. Finally, we established Wnd as a general modulator of cell death in *Drosophila* by showing that it is also required for ectopic Egr or Rho1 and loss of Scribble (Scrib)-induced cell death.

## Results and Discussion

### Wnd is essential for Rac1-induced cell death and JNK activation

**Figure 1:**
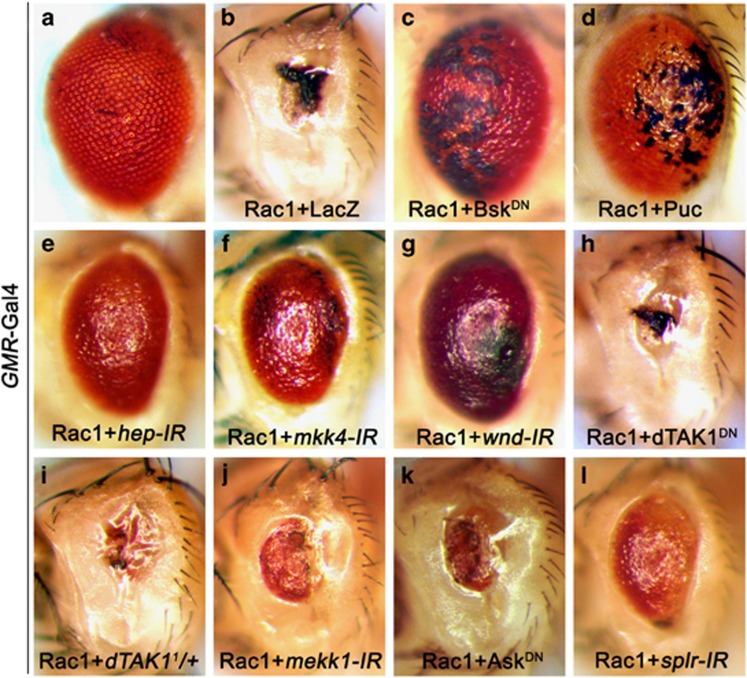
Wnd is essential for Rac1-induced small-eye phenotype. Light micrographs of *Drosophila* eyes are shown. Compared with the *GMR*-Gal4 control (**a**), *GMR*>Rac1-induced small-eye phenotype (**b**) was significantly suppressed by expression of Bsk^DN^ (**c**) or Puc (**d**), or RNAi-mediated knocking down of *hep* (**e**), *mkk4* (**f**) or *wnd* (**g**), and partially suppressed by knocking down *mekk1* (**j**), Ask1 (**k**) or *slpr* (**l**), but remained unaffected by expression of dTAK1^DN^ (H) or mutation in *dTAK1* (**i**)

**Figure 2:**
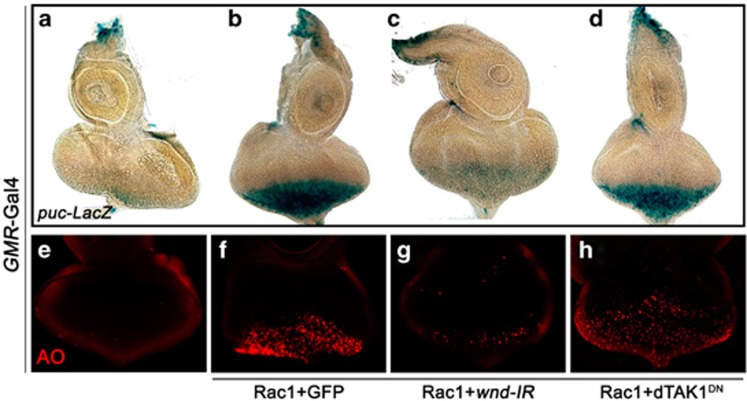
Wnd is required for Rac1-induced JNK activation and cell death. Light (**a–d**) and fluorescence (**e–h**) micrographs of *Drosophila* eye discs are shown. Compared with the *GMR*-Gal4 control (**a** and **e**), Rac1-induced upregulated *puc* transcription (**b**) and cell death (**f**) posterior to MF in third instar eye discs were suppressed by the expression of a *wnd* RNAi (**c** and **g**), but not that of dTAK1^DN^ (**d** and **h**)

Consistent with previous results that overexpression of the small GTPase Rac1 would affect eye development,^7, 14^ we found that expression of Rac1 under *GMR* promoter produced a complete eye loss phenotype (Figure 1b), resulting from extensive cell death posterior to the morphogenetic furrow (MF) in third instar eye discs (Figure 2f), as shown by acridine orange (AO) staining, a dye used to detect dying cells.^15^ In accordance with the genetic evidence that Rac1 regulates JNK-mediated dorsal closure,^10^ we found that blocking JNK activity by expressing a dominant negative allele of Bsk (Bsk^DN^) or the JNK phosphatase Puc could dramatically suppress Rac1-triggered eye loss phenotype (Figures 1c and d), although some pigment cells defects still remain. Furthermore, knocking down either of the two JNK kinases, Hemipterous (Hep) or MKK4, significantly suppressed Rac1-triggered no-eye phenotype (Figures 1e and f), indicating a critical role of JNK signaling in Rac1-induced cell death.

In *Drosophila*, upstream of Hep and MKK4 in the JNK pathway are five JNKKKs, including dTAK1, Slpr, Mekk1, Ask1 and Wnd. All of them, except Wnd, have been previously implicated in cell death.^16, 17, 18, 19, 20, 21^ In addition, dTAK1 plays a role in innate immunity,^22^ Slpr is required for dorsal closure,^7^ and Ask1 is involved in pigmentation.^23^ Wnd has been shown to play pivotal roles in regulating axon transportation, regeneration and degradation,^24, 25, 26^ but its role in cell death has remained unknown. We found *GMR*>Rac1-induced no-eye phenotype was slightly suppressed by knocking down *mekk1*, *Ask1* or *slpr* (Figures 1j and l), but remained unaffected by expressing a dominant negative form of dTAK1 (dTAK1^DN^) or mutation in *dTAK1* (Figures 1h and i), suggesting dTAK1 is dispensable for Rac1-triggered cell death. Consistent with previous studies, expression of dTAK1^DN^ almost completely suppressed *GMR*>Egr-induced small-eye phenotype (Supplementary Figures 1A and C),^20, 21, 27, 28^ suggesting dTAK1 is specifically required for Egr- but not Rac1-triggered JNK-dependent cell death. Intriguingly, we found that knocking down *wnd* dramatically suppressed Rac1-induced no-eye phenotype (Figure 1g). Consistently, Rac1-induced JNK activation (indicated by *puc-LacZ* staining^29^) and cell death in developing eye disc were also suppressed by knocking down *wnd* (Figures 2a–c and e–g), but remained unchanged by blocking dTAK1 activity (Figures 2d and h). Together, the above data demonstrate that *wnd* plays a major role in mediating Rac1-triggered cell death in *Drosophila*.

Rac1–JNK signaling is also known to play essential role in the process of dorsal and thorax closure during normal development.^10^ In accordance with previous study, we found knocking down *slpr* in the thorax by *pnr*-Gal4 produced a cleft phenotype (Supplementary Figures 2A and B),^7^ whereas depletion of *wnd* produced no obvious phenotype (Supplementary Figure 2C). Thus, Wnd appears dispensable for the thorax closure function of Rac1–JNK signaling.

### Wnd is physiologically required for JNK-mediated cell death

**Figure 3:**
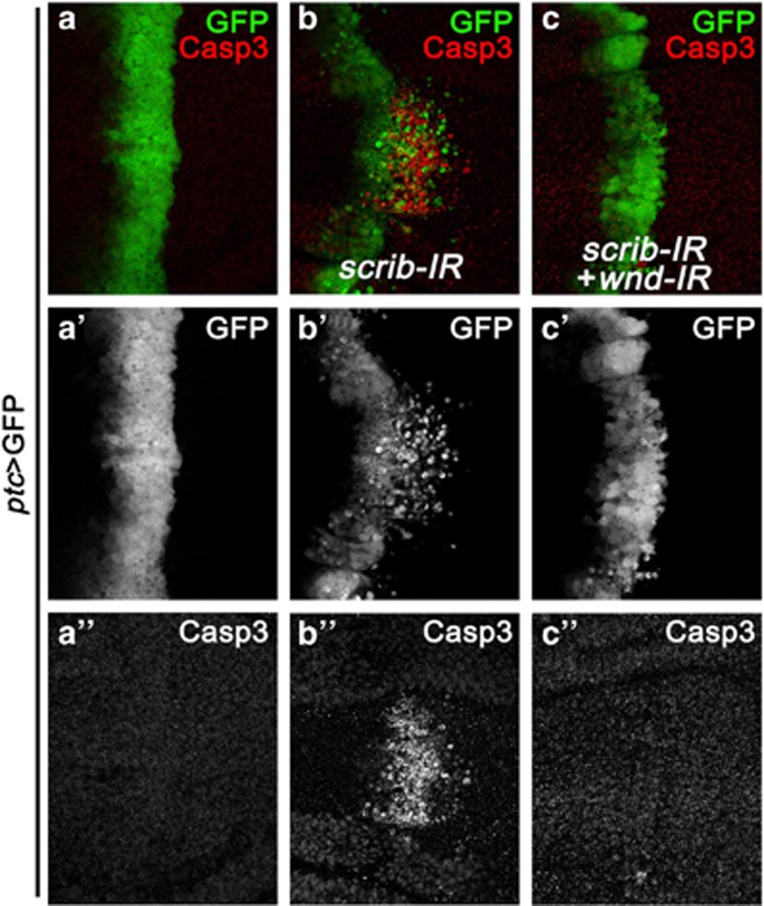
Wnd is required for loss of *scrib*-induced cell death. Fluorescence micrographs of *Drosophila* wing discs are shown. Compared with *ptc*-Gal4 control (**a–a**''), loss of *scrib*-induced cell death and invasion (**b–b**'') was strongly impeded by knocking down *wnd* (**c–c**'')

Correct establishment and maintenance of cell polarity are critical for development and tissue homeostasis. Loss of cell polarity results in JNK-dependent cell death and invasion in *Drosophila*.^30, 31, 32, 33, 34, 35^ To investigate whether *wnd* is required for the physiological functions of JNK signaling in development, we knocked down *scrib* along the anterior/posterior (A/P) compartment boundary in third instar larval wing discs by *patched* (*ptc*)-Gal4, and observed intensive cell death (indicated by cleaved Caspase 3 staining) and cell invasion into the posterior compartment (Figures 3b–b”). Depletion of *wnd* dramatically suppressed loss of *scirb*-induced cell death and invasion phenotypes (Figures 3c–c”), suggesting that Wnd also modulates the physiological functions of JNK signaling.

### Wnd induces JNK-mediated cell death

**Figure 4:**
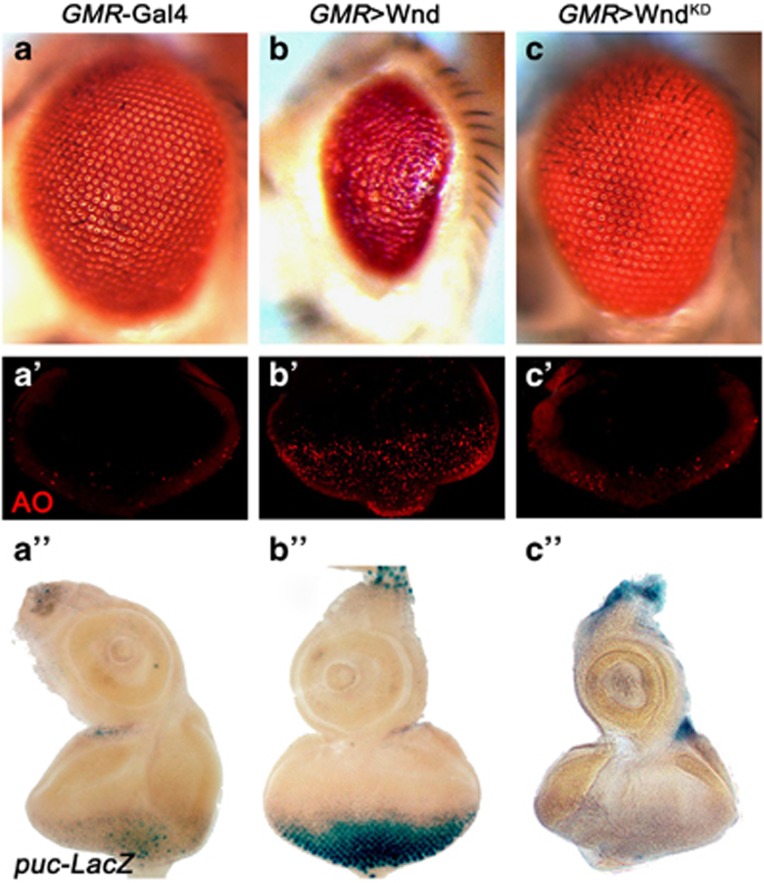
Wnd requires its kinase domain to induce JNK activation and cell death. Light micrographs of *Drosophila* eyes (**a–c**), eye disc (**a**''–**c**'') and fluorescence micrographs of eye discs (**a**'–**c**') are shown. Compared with the control (**a–a**”), expression of Wnd induced extensive cell death (**b**') and elevated *puc* transcription (**b**'') in eye discs, and produced a small-eye phenotype in adults (**b**), whereas expression of Wnd^KD^ produced no obvious phenotypes (**c–c**'')

**Figure 5:**
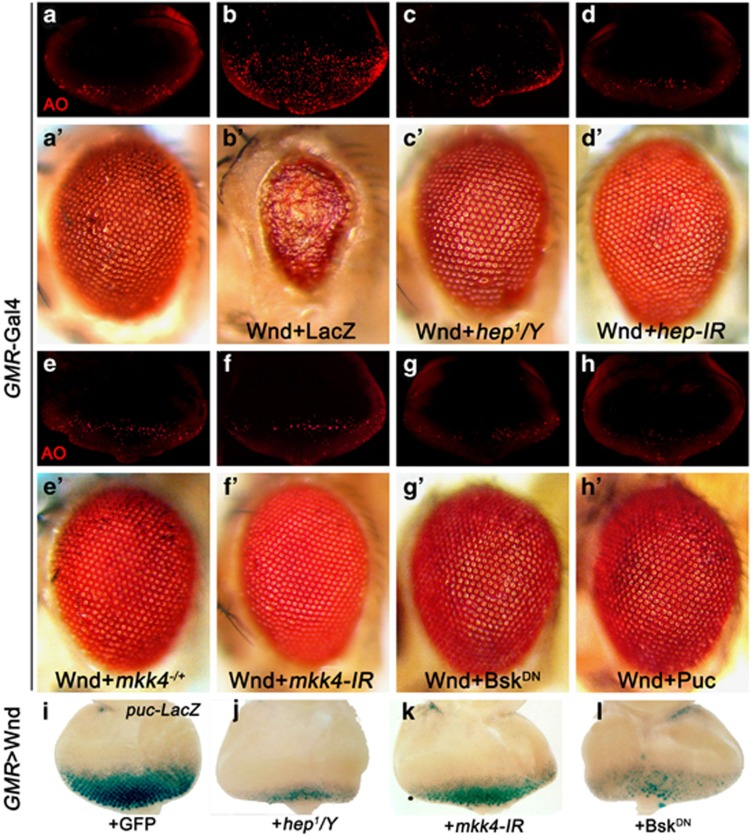
Wnd acts through MKK4 and Hep to induce JNK-dependent cell death. (**a–h**) Fluorescence micrographs of *Drosophila* eye discs (**a**–**h**) and light micrographs of adult eyes (**a**'–**h**') are shown. Compared with the control (**a**), Wnd-induced cell death and small-eye phenotype (**b**) could be strongly suppressed by loss of *hep* (**c** and **d**) or *mkk4* (**e** and **f**), or expression of Bsk^DN^ (**g**) or Puc (**h**). (**i–l**) Light micrographs of *Drosophila* eye disc are shown. Wnd-induced *puc*-LacZ expression (**i**) was impeded by loss of *hep* (**j**) or mkk4 (**k**), or expression of Bsk^DN^ (**l**)

Next, to examine whether Wnd is sufficient to induce JNK activation and cell death, we expressed Wnd in the developing eye under the *GMR* promoter, and observed a small-eye phenotype in the adults (Figure 4b). As expected, such phenotype could be suppressed by coexpression of a *wnd RNAi* (data not shown). In addition, Wnd prompts extensive cell death and JNK activation in third instar eye discs posterior to the MF, as indicated by AO staining (Figure 4b') and *puc*-LacZ expression (Figure 4b''), respectively. In contrast, a kinase-dead form of Wnd (Wnd^KD^) ^25^ fails to induce cell death and JNK activation in the eye disc, and produces a wild-type eye in the adults (Figures 4c–c''), suggesting the kinase domain is necessary for Wnd to induce JNK activation and cell death. Finally, Wnd-triggered JNK activation, cell death and small-eye phenotype is fully suppressed by coexpression of Bsk^DN^ or Puc (Figures 5g, g'), indicating Wnd triggers JNK-dependent cell death.

### MKK4 and Hep are both required for Wnd-induced cell death

Previous studies found DLK (Wnd ortholog in mammal) utilize MKK7 (Hep ortholog) but not MKK4 as a substrate in mammalian cells.^36^ To investigate whether Mkk4 or Hep is required for Wnd-induced JNK activation and cell death, we reduced their activities by mutations or RNAi expression. Intriguingly, loss of either *hep* or *mkk4* strongly blocked *GMR*>Wnd-induced cell death in eye discs (Figures 5c–f) and the small-eye phenotype in adults (Figures 5c'–f'), suggesting both MKK4 and Hep are necessary for Wnd-induced cell death *in vivo*. Consistently, both MKK4 and Hep are required for Wnd-triggered JNK activation, as loss of either gene strongly suppressed Wnd-induced *puc*-LacZ expression (Figures 5i–k). Collectively, these results imply that MKK4 and Hep might work together rather than in parallel, for instance in the same complex, to mediate Wnd-triggered JNK activation and cell death in *Drosophila*.

### Wnd is required for Egr-induced cell death and JNK activation

**Figure 6:**
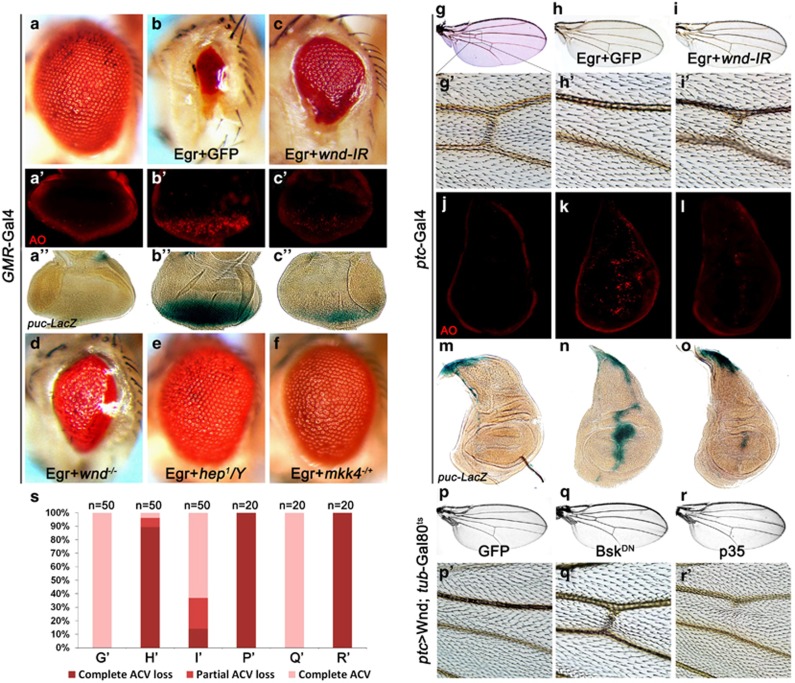
Wnd regulates Egr-induced cell death and JNK activation. (**a–f**) Compared with the *GMR*-Gal4 control (**a–a**”), Egr-induced small-eye phenotype (**b**), cell death (**b**') and *puc*-LacZ expression (**b**'') were suppressed partially by knocking down *wnd* (**c–c**''). The *GMR*>Egr small-eye phenotype was suppressed partially in *wnd* mutants (**d**, *wnd*^*1*^*/wnd*^*3*^), but near fully in hemizygous *hep* (**e**, *hep*^*1*^*/Y*) or heterozygous *mkk4* (**f**, *mkk4*^*G673*^/+) mutants. (**g–o**) Compared with the *ptc*-Gal4 control (**g**, **j** and **m**), Egr-triggered cell death (**k**) and JNK activation (**n**) in wing discs and the loss of anterior crossvein in adult wings (**h** and **h**') were suppressed by knocking down *wnd* (**i**, **i**', **l** and **o**). (**p–r**) Wnd-induced loss of anterior crossvein phenotype (**p** and **p**') was completely suppressed by the expression of Bsk^DN^ (**q** and **q**'), but not that of p35 (**r** and **r**'). (**s**) Quantification data of loss of anterior crossvein phenotype in (**g**', **h**', **i**', **p**', **q**' and **r**')

It has been reported that both MKK4 and Hep are required for cell death induced by Egr (Figures 6b, e and f), the *Drosophila* ortholog of TNF.^37^ Although dTAK1 has been previously implicated in Egr-induced JNK activation and cell death,^20, 38^ a potential role of Wnd in Egr–JNK signaling cannot be excluded. Indeed, we found that knocking down *wnd* partially suppressed *GMR*>Egr-induced cell death and JNK activation in eye discs (Figures 6a'–c' and a''–c''), and the small-eye phenotype in adults (Figures 6a–c). This suppression was further confirmed in *wnd* mutants (Figure 6d), suggesting Wnd also contributes to Egr-induced cell death. However, inactivation of dTAK1 almost fully blocked *GMR*>Egr-induced small-eye phenotype (Supplementary Figure 1C), suggesting dTAK1 is the major MAPKKK in Egr-induced JNK-dependent cell death. Furthermore, in accordance with the role of Rac1 in regulating JNK-mediated cell death, we found Rac1 is also required for *GMR*>Egr-induced small eye and cell death (Supplementary Figures 1B and E).

To investigate whether Wnd is required for Egr-induced cell death in a nontissue-specific manner, we characterized the genetic interaction between Wnd and Egr in the developing wing. Expression of Egr driven by *ptc*-Gal4 triggers cell death in the wing disc and generates a loss of anterior crossvein (acv) phenotype in the adult wing, both of which were strongly suppressed by loss of *wnd* (Figures 6g–l, g'–i' and s). Furthermore, *ptc*>Egr-induced *puc*-LacZ expression in the wing disc was also suppressed by depletion of *wnd* (Figures 6m–o). Together, these results demonstrate that Wnd is required for Egr-induced JNK activation and cell death in wing development.

Furthermore, expression of Wnd driven by *ptc*-Gal4 recapitulates the loss-of-acv phenotype of *ptc*>Egr (Figures 6p, p' and s). As *ptc*>Wnd results in lethality at larva stage, we used *tub*-Gal80^ts^ expressing a temperature-sensitive form of the Gal4 inhibitor Gal80 (Gal80^ts^) to block Gal4 activity at low temperature (18 °C), and to unchain the inhibition at high temperature (29 °C).^39^ Interestingly, *ptc*>Wnd-induced loss-of-acv phenotype was fully suppressed by inactivation of JNK (Figures 6q, q' and s), but remained unaffected by expression of p35 that blocks caspase's activity (Figures 6r, r' and s). These data are consistent with our previous report that JNK signaling induces caspase-independent cell death.^27^

### Wnd acts in parallel with dTAK1 in the TNF–JNK signaling pathway

**Figure 7:**
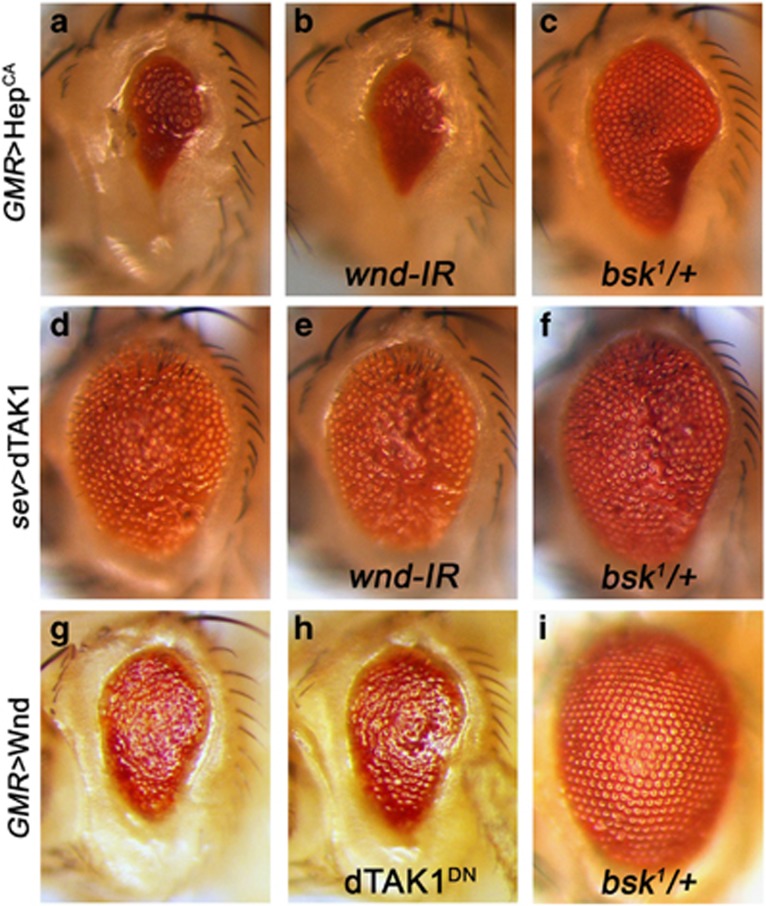
Wnd acts in parallel with dTAK1 in Egr–JNK pathway. Light micrographs of *Drosophila* eyes are shown. The small-eye phenotype of *GMR*>Hep^CA^ (**a**) and the rough eye of *sev*>dTAK1 (**d**) was not suppressed by knocking down *wnd* (**b** and **e**), but was significantly suppressed in heterozygous *bsk* mutants (**c** and **f**). Conversely, *GMR*>Wnd-induced small-eye phenotype (**g**) was not affected by blocking dTAK1 activity (**h**), but was dramatically suppressed in heterozygous *bsk* mutants (**i**)

The above results suggest that Wnd may act as a novel component in the TNF–JNK signaling pathway. To further genetically map Wnd in this pathway, we performed epistasis analysis between Wnd and dTAK1 or Hep. Consistent with previous data, expression of a constitutive activated form of Hep (Hep^CA^) in the developing eye under *GMR* promoter induced JNK-mediated cell death and resulted in a small-eye phenotype (Figure 7a).^27, 32^ This phenotype could not be suppressed by loss of Wnd (Figure 7b), consistent with our genetic data that Hep is required for Wnd-induced cell death (Figures 5c and d). Furthermore, we found dTAK1-triggered rough-eye phenotype (Figure 7d)^34^ remained unaffected by the loss of Wnd (Figure 7e). Conversely, blocking dTAK1 activity could not suppress Wnd-induced small-eye phenotype as well (Figures 7g and h). As a positive control, the eye phenotype induced by ectopic expression of Hep^CA^, dTAK1 or Wnd was significantly suppressed by a mutation in one copy of endogenous *bsk* (Figures 7c, f and i; Supplementary Figure 3). Together, these results indicate that Wnd acts in parallel with dTAK1 in regulating JNK-mediated cell death.

### Wnd is required for Rho1-induced cell death

**Figure 8:**
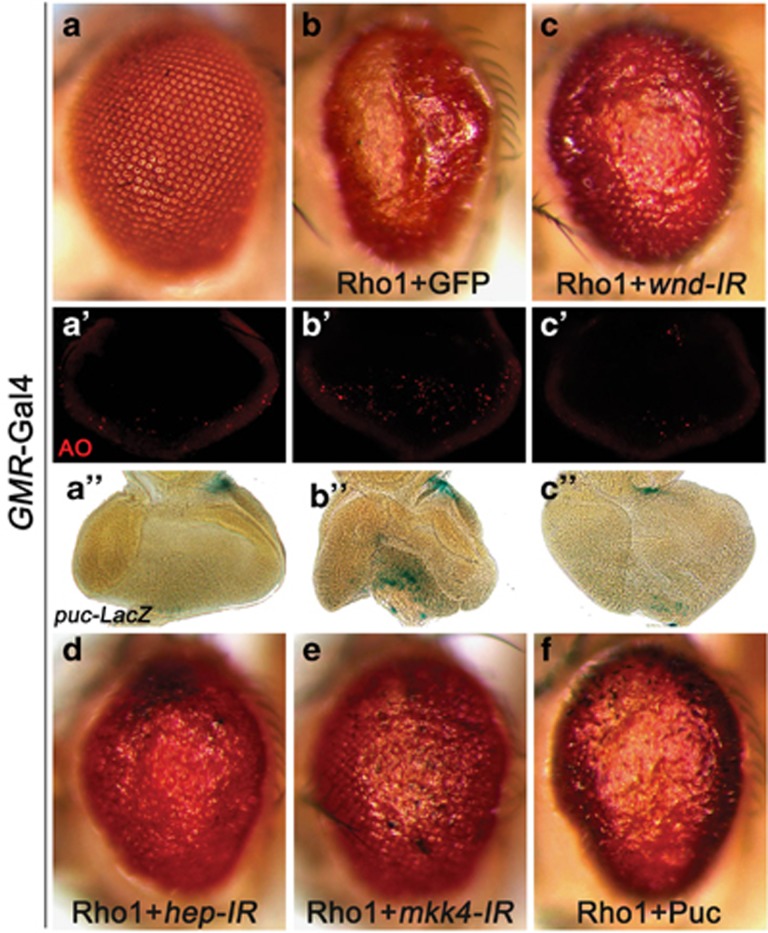
Wnd is required for Rho1-induced cell death and JNK activation. Compared with the control (**a–a**''), *GMR*>Rho1-triggered cell death (**b**') and *puc*-LacZ expression (**b**'') in eye discs and small-eye phenotype (**b**) were significantly suppressed by knocking down *wnd* (**c–c**''). The *GMR*>Rho1 small-eye phenotype was also suppressed by knocking down *hep* (**d**) or *mkk4* (**e**), or expression of Puc (**f**)

Apart from Rac1, another Rho GTPase family member Rho1 has been implicated in cell death and neurodegeneration.^18, 40^ In accordance with these findings, we found ectopic Rho1 expression driven by *GMR*-Gal4 resulted in increased cell death and JNK activation in third instar eye discs and produced a small rough-eye phenotype in adults (Figures 8b–b''). These phenotypes were suppressed by knocking down *wnd* (Figures 8c–c''), suggesting Wnd is also required for Rho1-induced JNK activation and cell death. Intriguingly, loss of *wnd* fully suppressed Rho1-induced JNK activation, cell death and reduced eye size, but not the rough-eye phenotype (Figures 8c–c''), suggesting Rho1-induced eye roughness is likely independent of JNK signaling. Consistent with this explanation, blocking JNK activity by knocking down *hep* or *mkk4*, or expressing Puc, was able to suppress the reduced size, but not the roughness, of *GMR*>Rho1 adult eyes (Figures 8d–f).

## Materials and Methods

### *Drosophila* stocks and genetics

All stocks were raised on standard *Drosophila* media and crosses were performed at 25 °C unless otherwise indicated. For experiments involving *tub*-Gal80^ts^, flies were raised at 18 °C to restrict Gal4 activity for 5–6 days, then shifted to 29 °C for 2 days to inactivate Gal80^ts^. The following stocks were used: *GMR*-Gal4, *ptc*-Gal4, *sev*-Gal4, *UAS*-GFP, *UAS*-Rac1 (6680), *UAS*-Rho1 (7334), *UAS*-LacZ (3956) and *wnd*^*Exel6135*^ (7614, EP line use for overexpression), all obtained from the Bloomington Stock Center (Bloomington, IN, USA), *UAS-Rac1-IR* (2248R-1)^43^ obtained from National Institute of Genetics (NIG, Mishima, Japan), *UAS*-Wnd^KD^, *wnd*^*1*^, *wnd*^*3*^ (gifts from Aaron DiAntonio, St. Louis, MO, USA), *UAS*-Ask1^DN^ (gift from Masayuki Miura, Tokyo, Japan), *hep*^*1*^, *UAS*-Egr, *UAS*-dTAK1, *UAS*-dTAK1^DN^, *UAS*-Bsk^DN^, *UAS-hep-IR*, *UAS*-Puc, *puc*^*E69*^,^44^ *bsk*^*1*^,^34^ *UAS*-Hep^CA^, *dTAK1*^*1*^,^27^ *UAS*-*wnd-IR*,^24^ *UAS-MKK4-IR*,^43^ *mkk4*^*G673*^,^37^ *UAS-slpr-IR*^18^ and *UAS-mekk1-IR*,^45^ as previously described.

### Immunostaining

Third instar larvae wing discs were fixed in freshly made 4% paraformaldehyde for 15 min and washed 3 times with 1 × PBS, then stained using rabbit anti-active Caspase 3 (1 : 200) (Cell Signaling Technology, Danvers, MA, USA). Secondary antibody was anti-rabbit-Cy3 (1 : 1000, Jackson Immunochemicals, West Grove, PA, USA).

### X-gal staining

Eye and wing discs were dissected from third instar larvae in PBST (1 × PBS pH 7.0, 0.1% Triton X-100) and stained for *β-*galactosidase activity.

### AO staining

AO staining was done as previously described.^33^ Briefly, eye or wing discs were dissected from late third instar larvae in PBST and incubated in 1 × 10^−5^ M AO for 5 min at room temperature before imaging.

## Conclusions

We have uncovered Wnd as a crucial regulator of JNK-mediated cell death in *Drosophila*. Specifically, our genetic epistasis analysis established Wnd as a novel cell death modulator downstream of Rac1, Egr, Rho1 and loss of *scrib*. Furthermore, we show that Wnd is sufficient to induce JNK-dependent cell death through both MKK4 and Hep, and this is different from previous mammalian study that DLK utilize only MKK7 as its substrate.^36^ Our finding also clarifies the independent roles of Wnd and the well-known JNKKK dTAK1 in regulating JNK-mediated cell death. Whereas dTAK1 is required only for Egr- but not Rac1-triggered cell death, Wnd plays crucial roles in both situations. Furthermore, Wnd and dTAK1 act in parallel to regulate JNK-dependent cell death upstream of MKK4 and Hep. Besides the established role in cell death, the *Drosophila* JNK pathway is also required for cell migration and tumor metastasis.^2, 34, 41^ Consistent with this notion, loss of DLK, the mammalian ortholog of Wnd, results in delayed radial migration of neuronal cells.^42^ Therefore, a potential role of Wnd in regulating cell migration and tumor metastasis is worth further investigation.

## Abbreviations

PCD: programmed cell death
JNK: c-Jun N-terminal kinase
A/P: anterior/posterior
AO: acridine orange
