# Supplementary material for: Wallenda regulates JNK-mediated cell death in Drosophila
Source: Cell Death Dis. 2015 May 7;6(5):e1737–. doi: 10.1038/cddis.2015.111 (PMC4669691; doi:10.1038/cddis.2015.111)
Supplement: Supplementary Information [file cddis2015111x1.docx]

**Supplementary Information:**

**Wallenda Regulates JNK-Mediated Cell Death in *Drosophila***

Xianjue Ma, Wenyan Xu, Di Zhang, Yang Yang, Wenzhe Li and Lei Xue

Supplementary Figure 1

Supplementary Figure 2

Supplementary Figure 3

**Ma et al., Supplemental Fig. 1**


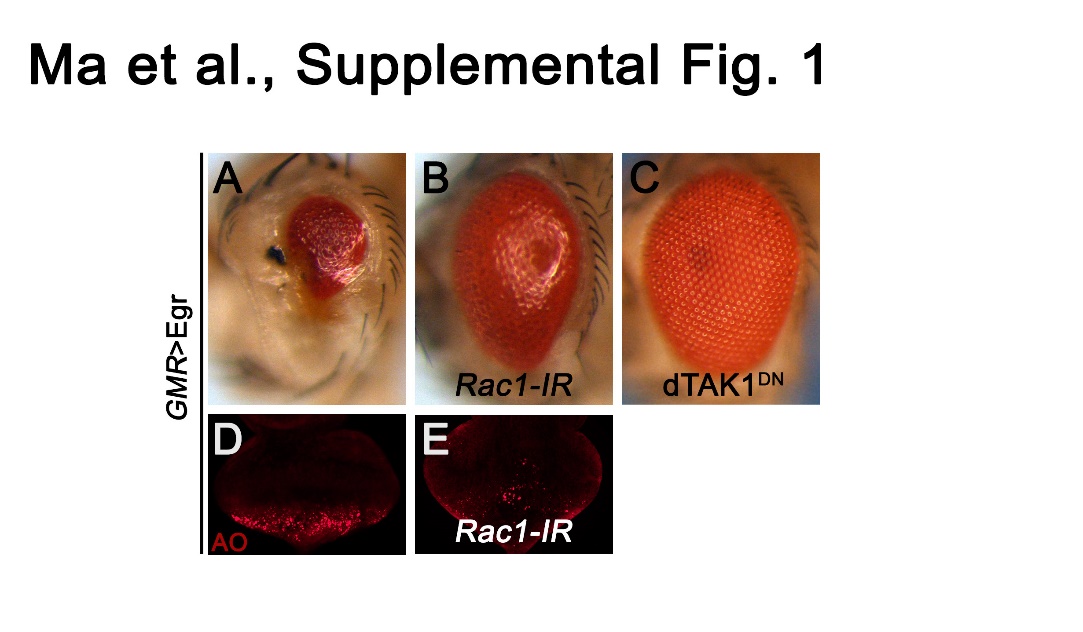


**Fig S1. Rac1 and dTAK1 are required for *GMR*>Egr induced cell death.**

Light (A-C) and fluorescence (D-E) micrographs of *Drosophila* eye discs are shown. Ectopic Egr expression induced small eye phenotype (A) and cell death (D, indicated by AO staining) were both significantly suppressed by reducing *Rac1* activity (B and E). Expression of a dominant negative form of dTAK1 almost completely suppressed *GMR*>Egr small eye (C).

**Ma et al., Supplemental Fig. 2**


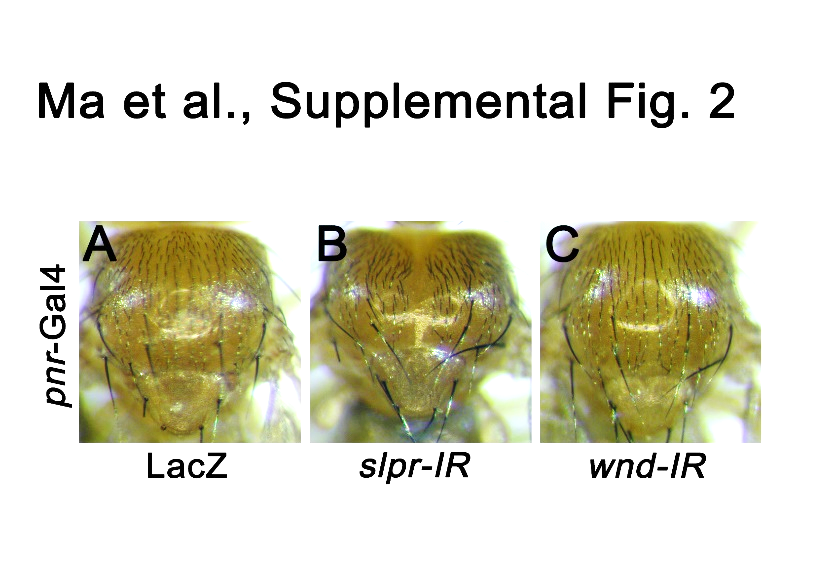


**Fig S2. Wnd is dispensable for thorax closure.**

Light micrographs of *Drosophila* thorax are shown. Compared with the *pnr*-Gal4 control (A), depletion of *slpr* in the thorax produced a cleft phenotype (B), while loss of *wnd* generated no obvious phenotype (C).

**Ma et al., Supplemental Fig. 3**


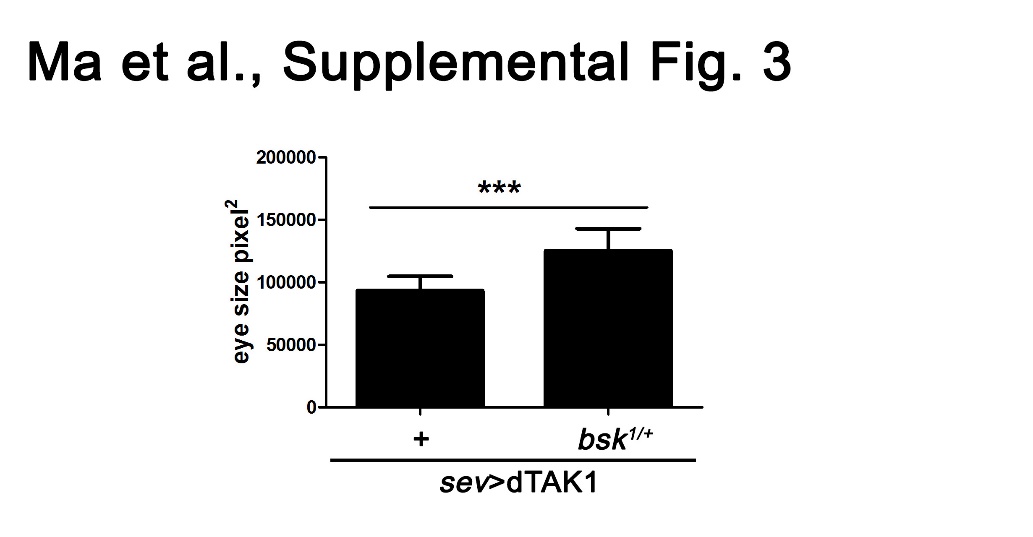


**Fig S3. JNK signaling is required for *sev*>dTAK1 induced small eye.**

Quantification analysis for data in Fig. 7D and F. Student’s *t* test was used, ***: *p*<0.001 (mean + SD, n = 8).
